# Hepatocellular Carcinoma: Understanding the Inflammatory Implications of the Microbiome

**DOI:** 10.3390/ijms23158164

**Published:** 2022-07-25

**Authors:** Ahamed A. Khalyfa, Shil Punatar, Alex Yarbrough

**Affiliations:** 1 Department of Internal Medicine, Franciscan Health Olympia Fields, Olympia Fields, IL 60461, USA; shilpunatar@gmail.com; 2 Department of Gastroenterology, Franciscan Health Olympia Fields, Olympia Fields, IL 60461, USA; ayarbrough22@gmail.com

**Keywords:** hepatocellular carcinoma, NAFLD, hepatitis B, hepatitis C, alcoholic liver disease, single cell

## Abstract

Hepatocellular carcinoma (HCC) is the third leading cause of cancer-related death worldwide. It is well known that repeated inflammatory insults in the liver can cause hepatic cellular injury that lead to cirrhosis and, ultimately, hepatocellular carcinoma. Furthermore, the microbiome has been implicated in multiple inflammatory conditions which predispose patients to malignancy. With this in mind, we explore the inflammatory implications of the microbiome on pathways that lead to HCC. We also focus on how an understanding of these underlying inflammatory principles lead to a more wholistic understanding of this deadly disease, as well as potential therapeutic implications.

## 1. Introduction

Hepatocellular carcinoma (HCC) is the most common primary malignant tumor of the liver and is the third leading cause of death from cancer worldwide. It has been well established that inflammation plays a central role to the pathogenesis of HCC with a typical sequence incorporating a chronic hepatic inflammatory insult, followed by liver fibrosis and cirrhosis, and, ultimately, carcinogenesis. There are multiple disease processes which feed into this paradigm, including non-alcoholic steatohepatitis (NASH), alcoholic fatty liver disease, and chronic viral hepatitis. Specifically, immune dysregulation involving infiltration of myeloid cells, tumor associated macrophages, and tumor associated neutrophils is considered a hallmark mechanism which leads to tumorigenesis [1]. In recent years, the study of the tumor microenvironment which includes inflammatory cells, stromal cells, the extracellular matrix and microbiota has taken center stage with respect to understanding of various malignancies. The microbiome specifically has been implicated in multiple cancers, including colon cancer, breast cancer, and lung cancer [2]. Although the study of the microbiome may present some disadvantages, such as technical difficulties in ruling out potential external bacterial contamination, new technologies, such as metagenomics and microbiome shotgun sequencing, help minimize these errors. In recent years, the microbiome has been shown to play an instrumental role in the inflammatory pathogenesis of multiple malignancies through its impact on the tumor microenvironment. In this paper, we explore the inflammatory role that the microbiome poses on the pathogenesis of HCC. We examine inflammatory implications on conditions which may lead to chronic liver disease and, ultimately, the development of HCC. We also explore how an understanding of these inflammatory mechanisms lead to novel treatment modalities.

## 2. Role of Microbiome on Inflammatory Processes in Conditions Which Lead to Chronic Liver Disease

### 2.1. Alcoholic Liver Disease

Although we discuss the broad category of alcohol use and its role on inflammation, microbiota changes, and potential for carcinogenesis, we focus on alcohol-related liver changes as a whole. Several changes are induced along various stages of liver damage, commonly referred to as the 5-Hit Hypothesis of Alcoholic Liver Disease, but our referenced mechanisms will convey the mechanisms as a whole. Within the stages of alcohol liver disease, we look at the changes induced from alcohol dehydrogenase and mitochondrial DNA leading to alcoholic fatty liver, with then its transformation to steatohepatitis, thirdly with transition to a more severe injury with alcoholic hepatitis, followed by transition to cirrhosis and completed with the fifth stage of hepatocellular carcinoma [3].

In discussing the role of alcohol-induced microbiota changes leading to HCC, we primarily consider two factors of necessity to be fulfilled: excessive alcohol consumption of over 20 g daily in females and 30 g daily in males, with the second being an increased chronicity of consumption [4]. These excessive and chronic changes are imposed upon the natural gut microbiome, which demonstrates a predominant prevalence of seven primary groups of micro-organisms: *Firmicutes, Bacteroidetes, Actinobacteria, Fusobacteria, Proteobacteria, Verrucomicrobia*, and *Cyanobacteria* [5]. Using pre-clinical models, gut flora of mice who were provided alcohol demonstrated small intestinal bacterial overgrowth, with dysbiosis in the cecum. Overall, a reduction in *Firmicutes* phyla, with an abundance of *Bacteroidetes* and *Verrucomicrobia,* was demonstrated. Furthermore, the prevention of alcohol-induced dysbiosis was shown in *Lactobaccilus* supplemented models [6]. In human models, there was a noted increase in inflammatory active microbiota with increased manifestation of *Proteobacteria*, along with *Firmicutetes* and *Colstridia*, with lower concentrations of the protective *Faecalibacterium* [6].

A major consideration of inflammation induced microbiome changes is in mechanisms of oxidative stress and intestinal permeability, colloquially known as the ‘leaky gut.’ With this, studies have demonstrated alcohol consumption to lead to upregulation of CYP2E1 dependent reactive oxygen species products, with accumulation in the liver leading to oxidative stress. Thereafter, structural and functional changes are imposed with eventual carcinogenesis [7]. Oxidative stresses also lead to lipid peroxidation products, such as malondialdehyde and 4-hydroxy-2-nonenal, which have the capacity to alter the gut microbiome and upregulate creation of endotoxins, as well as induce changes in the p53 gene, ultimately leading to HCC formation [3,8]. As the gut connects to the liver via portal and biliary circulation, there is direct transfer of gut derived products directly impacting liver pathophysiology. In consideration of endotoxins, alcohol liver disease leads to a decrease in bacterial diversity, with increased Enterobacteriaceae and *Proteobacteriaceae* led endotoxin production [9]. Commonly seen in alcoholic liver disease is reduced expression of lectins, leading to bacterial overgrowth and translocation [10]. With increased endotoxin production, there is further intestinal inflammation, leading to activation of TNF-alpha receptor signaling, and subsequent permeability of endotoxins to the liver [11]. This is augmented by nitration and oxidation of tubulin, leading to microtubule damage and subsequent activation of NF-kB signaling, leading to tight junction disruption [12].

Bacterial dysbiosis is present even in early stages of alcoholic liver disease, in which we notice the role of decreased anti-microbial molecules, such as Reg3b and Reg3g lectin, increased mucosal associated bacteria, and bacterial translocation to mesenteric lymph nodes [13]. In consideration of dysbiosis, we look at the trends and downstream changes induced by metabolomics pathways. In regard to gut microbiota in HCC, in human and animal testing, there has been a disturbance in the ratio of *Lactobacillus* to *Bifidobacterium*, with increased pathogenic bacteria, primarily *Proteobacteria* and *Bacilli* [14]. This arises as *Lactobaccilus* aids in the integrity of gut epithelium, however with alcoholic liver disease leading to decreased long chain fatty acids, with a subsequent decrease in *Lactobaccilus* growth.

In consideration of gut microbiota alterations in HCC pathogenesis, this understanding also points to potential therapeutic options. For one, antibiotics, such as rifaximin and norfloxacin, have demonstrated increased survival in patients with HCC due to their role in microbiota regulation. Along these lines are the positive role of probiotic compounds in aiding to restore natural bacterial diversity [15].

### 2.2. NAFLD

As previously mentioned, the prevalence of hepatocellular carcinoma makes it a keen topic of consideration, understanding, and study, and when considered within the context of non-alcoholic fatty liver disease, it is becoming one of the largest threats in end-stage liver diseases. Currently, NAFLD serves as the second most common cause of liver cancer, with studies demonstrating a potential doubling of cases by the year 2030, likely becoming the most common cause of hepatocellular carcinoma in the future [16]. As discussed with alcoholic liver disease, the progression and spectrum of NAFLD is traditionally described in four main stages, with distinct pathophysiological changes along each variation, each imposing a risk to transition directly to hepatocellular carcinoma. NAFLD can be broken into stages of non-alcoholic simple fatty liver, non-alcoholic steatohepatitis, hepatic cirrhosis, and ultimately hepatocellular carcinoma. Here, we describe the overview of microbiological and inflammatory changes induced along each transitionary phase.

When compared to the composition of gut microbiota in healthy individuals, NAFLD patients demonstrate increased concentrations of *Blautia, Dorea, Lactobaccilus, Clostridium, Allisonella, Parabacteroides*, and *Escherichia* species, with decreased concentrations of *Oscillospira, Corprococcus, Faecalibacterium*, and *Bifidobacterium* species [16]. With these compositional changes also come changes similar to those seen in alcoholic liver disease, such as the ‘leaky gut’, metabolite derived complications and hepatic inflammation.

In clinical studies and animal trials it has been found that intestinal permeability is mediated to some extend by various microbial factors, with decreased expression of tight junction proteins, namely zonula occuldens-1 (ZO-1) [17]. In NAFLD populations, microbial derangements create decreased populations of *Bifidobacterium* species, certain Lactobaccili and *A. muciniphilia*, known to induce ZO-1 production for gut barrier integrity [18]. Production of microbes, such as *Desulfovibrio* species, also produced concentrations of hydrogen sulfide, with its genotoxicity leading to intestinal permeability [19]. Namely, studies have demonstrated increased *E. coli* growth in HCC patients, as well as *Bacteroiides* and *Ruminococcaceae* species, with decreased levels of *Akkermensia* and *Bifidobacterium* species [16].

With increased gut permeability, hepatotoxic microbe-derived molecules exhibit increased entry, leading to further hepatic inflammation. The increased permeability to products such as lipopolysaccharide has demonstrated further downstream inflammation through activation of Toll-like receptor 4, including activation of Kupffer cells, hepatic stellate cells, and sinusoidal endothelial cells. Specifically, in Kupffer cells, TLR4 signaling via myeloid differentiation primary response 88 increases TNF-alpha activation with reactive oxygen species production and further hepatic inflammation [20]. In this, transition from NAFLD to HCC follow a similar process. The dysbiosis and intestinal permeability lead to pathogen-associated molecular protein (PAMP) and microbiota metabolites influx in the liver, with subsequent hepatic inflammation and perturbed metabolic homeostasis. PAMPs aid in activation of TLR, with the induction of cytokine and chemokine production, allowing for immune cell infiltration into the liver. Activation of hepatic stellate cells also occurs, inducing epireulin to further fibrosis [21,22]. With cytokine secretion, such as TNF-alpha and interulekin-8, there is also downstream production of IL-1 β by Kupffer cells. IL-1 β promotes lipid accumulation and apoptosis of hepatocytes with subsequent steatosis and inflammation with activation of further hepatic stellate cells, leading to fibrogenic mediators and the development of HCC [23]. Variations in gut microbiota also provoke alterations in bile acid metabolism, leading to higher levels of deoxycholic acid with activation of its farnesoid X receptor and secretion of inflammatory and tumor promoting factors in the liver via hepatic stellate cells, further promoting development of HCC [24,25].

**Figure 1:**
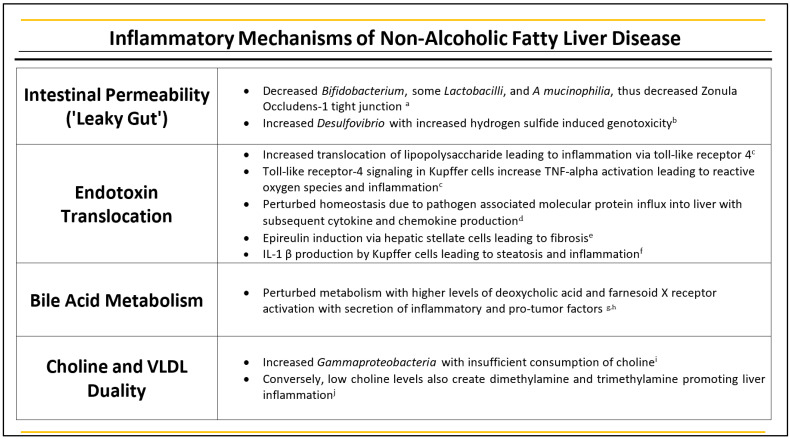
Table depicting the role of leaky gut, endotoxin translocation, bile acid metabolism, choline, and vLDL duality in NAFLD. ^a^ Lam et al., 2012 [18], ^b^ Lam et al., 2015 [19], ^c^ Kolodziejczyk et al., 2019 [20], ^d^ Dapito et al., 2012 [21], ^e^ Schwab et al., 2020 [22], ^f^ Raza et al., 2019 [23], ^g^ Yoshimoto et al., 2013 [24], ^h^ Takahashi et al., 2018 [25], ^i^ Spencer et al., 2011 [28], ^j^ Chu et al., 2018 [29].

Aside from variations in the composition of gut microbiota, concentrations alone can contribute to the carcinogenicity and pathogenesis of NAFLD to HCC. The disease entity known as small intestinal bacterial overgrowth (SIBO) carries a direct relationship with severity of liver disease severity, of prevalence of between 39% and 85% in NAFLD patients [19]. SIBO demonstrates increased LPS secretion and inflammation, with expression of TLR4 exhibiting release of interleukin-8 increased inflammation, a shared process with dysbiosis in general [26]. Although NAFLD, in terminology, refers to a disease process without exogenous ethanol use, studies also point to hepatotoxicity by endogenously synthesized ethanol. Alcohol is constantly produced by intestinal microbiota with studies demonstrating higher levels in diets rich in sugar-containing foods [27]. In NAFLD patients, microbiota changes have been studied with documented variations in *Proteobacteria*, *Enterobacteriaceae*, and *E. coli* with these microbiota changes allowing for augmentation of ethanol production, compared to healthy individuals [26]. This, in turn, suggests that endogenous alcohol production via microbial derangements in NAFLD serve as potential hepatoxins in the transition from NAFLD to end-stages of liver disease and HCC. Recent studies have also illuminated the multi-dimension role and balance of choline and its effect on hepatic homeostasis. Choline aids in lipid and VDLD metabolism, with it being a constituent of cell and mitochondrial membranes. In subjects with low dietary choline, it has been noted they demonstrate variable populations of *Gammaproteobacteria*, which are not present in sufficient levels of choline consumption, demonstrating that choline directly plays a role in microbiota composition [28]. On the other hand, gut microbiota also converts dietary choline to dimethylamine and trimethaylamine, which are absorbed through microvilli to portal circulation and promote liver inflammation. Interestingly, even studies with low choline levels have been noted to promote fatty liver disease [27]. This duality lends to yield further exploration into the balance and role of choline metabolism on gut microbiota induced transformation from NAFLD to HCC (Figure 1).

### 2.3. Hepatitis B

In consideration of changes to microbiota in Hepatitis B (HBV), HBV carriers have demonstrated significant variations and alterations to their gut microbiota in comparison to healthy hosts. Primarily, there is a noticeable increase in the growth rate of *E. coli* (Mohamadkhani). Furthermore, there have been demonstrated increases in *Enterobacteriaceae*, *Enterococcus faecalis*, and *Faecalibacterium* (Mohammadkhani). Conversely, a lower quantity of species, such as *Lactobaccilis*, *Pediococcus*, and *Weisella,* have been reported [30]. Further noted is the trend seen in various inflammation-based microbial changes leading to HCC in that there is variation in the *Bifidobacteria*/*Enterobacteriaceae* ratio [29]. As previously described, the advantage of intestinal bacterial in healthy hosts is the production of short chain fatty acids, secondary bile acids, and the production of butyrate by beneficial colonic bacteria to act as colonocyte energy. In patients with HBV, chronicity has demonstrated a decrease in the production of polyunsaturated lipids due to disintegrity of the intestinal barrier, with lower levels of bile acids, and alterations on butyrate production [30].

The overproduction of lipopolysaccharide as an endotoxin from Gram-negative bacteria has also been studied in HBV patients, with downstream effects on TLR4 signaling [31]. LPS stimulates inflammation in TLR4 signaling pathways, leading to recognition and expression of innate immune responses and subsequent expression of inflammatory responses and proinflammatory cytokines [31]. Furthermore, TLR4 activation leads to inhibition of tumor necrosis factor alpha, and as TLR4 is expressed on Kupffer cells and hepatic stellate cells, this receptor becomes a significant mediation in hepatic fibrosis [32,33].

Interestingly, some studies have demonstrated a potential beneficial role of microbiome changes in HBV, however citing that the HBV in itself will lead to a host’s ultimate demise. For example, Milosevic et al. report HCC patients with HBV show higher species richness with anti-inflammatory bacteria, such as *Faecalibacterium*, and others, along with fewer pro-inflammatory bacteria, such as *Enterococcus*, however HBV infection leads to progressive decline compared to healthy subjects [34]. To further this consideration, in patients with HCC due to HBV or HCV there were higher amounts of anti-inflammatory bacteria and lower pro-inflammatory bacteria in comparison to HCC patients who were HBV/HCV negative [29,35].

### 2.4. Hepatitis C

With consideration of HBV, HCV also poses a significant risk as a global health problem as it leads to liver fibrosis and cirrhosis, with 1–4% of patients developing HCC each year [36]. Although substantial literature does not yet exist on HCV induced microbiota changes, published accounts document alteration in beneficial micro-organisms, predominance of harmful micro-organisms, and alteration of total microbial diversity [36].

Specifically, in HCV, it has been documented that microbiome dysbiosis is contributed to by IgA produced by gastric B-lymphocytes infected by HCV [34]. Further, in HCV infection, *Clostridiales* is noted to decrease with the increase in *Lactobaccilus* and *Streptococcus* genera. Moreover, *Bacteroidetes* phyla are increased while phyla *Firmicutes* are decreased [37]. By published accounts, the gut microbiota of HCV patients demonstrated more *Prevotella* and *Faecalibacterium* with less *Acinetobacter* and *Veillonella* in comparison to healthy individuals [38]. Additionally, seen is the overgrowth and enrichment of *S. salivarius*, which is known to down-regulate immune responses, specifically in the setting of HCV-induced advanced liver cirrhosis and HCC, suggesting its role in HCC development and progression [39].

Similar to the trends seen in HBV and NAFLD, and alcoholic liver disease states, metabolic dysfunction of bile acids has demonstrated overgrowth of pro-inflammatory bacteria in HCV hosts [40]. Further, both *Ruminococcaecae* and *Lachnospiraceae*, known to belong to major SCFA producing families, are also decreased in HCV patients, with subsequent gut dysbiosis and inhibited inflammation suppression [37].

**Figure 2:**
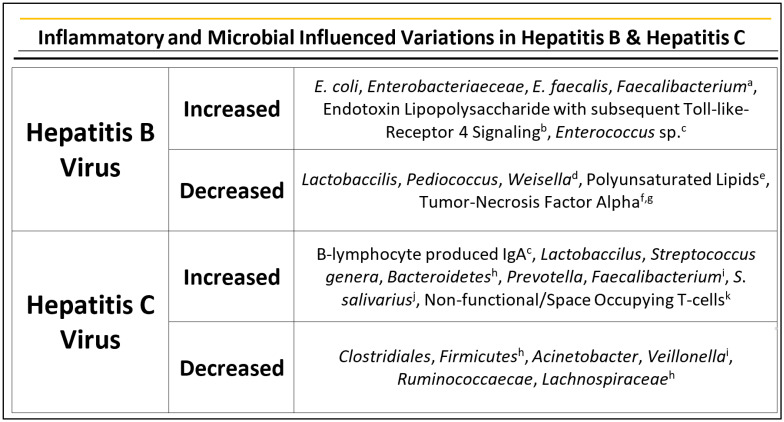
Upregulation and downregulation of microbes and their factors related to hepatitis B and C. ^a^ Mohamadkhani et al., 2018 [32], ^b^ Palsson McDermott et al., 2014 [33], ^c^ Milosevic et al., 2019 [36], ^d^ Lu et al., 2011 [30], ^e^ Wang et al., 2017 [31], ^f^ Ma et al., 2015 [34], ^g^ Roderburg et al. 2014 [35], ^h^ El-Mowafy et al., 2021 [39], ^i^ Aly et al., 2016 [40], ^j^ Qin et al., 2018 [41]., ^k^ Giannakoulis et al., 2021 [43].

While discussing microbial dysbiosis induced by HCV, we also shed light on the unique role of senescence induced by HCV. In chronic states, HCV in itself as an alteration of gut microbiota causes liver inflammation with acceleration of telomere shortening, leading to replication changes predisposing to HCC [41]. In patients with chronic HCV infection, there is a noticeable increase in non-functional T-cells, which occupy vital space where immune cells can be located in the liver and functioning T-cells could assist in clearance of pre-malignant hepatocytes [41]. HCV also suppresses premature hepatocellular cells by imposing oxidative stress leading to cellular damage via DNA alterations leading to HCC [42] (Figure 2).

Although the aforementioned conditions of alcoholic liver disease, NAFLD, hepatitis B, and hepatitis C have been shown to increase the risk of HCC, it is important to note that these conditions are usually associated with other risk factors and are not mutually exclusive. For example, alcohol use is associated with use of other substances, such as tobacco, as well as conditions such as obesity and diabetes mellitus which are associated with NAFLD. With this in mind, it is important to critically analyze studies regarding risk factors of HCC in order to effectively identify independent risk factors.

## 3. Role of the Microbiome on Inflammatory Processes in HCC

The implications of the microbiome on liver cancer have garnered great interest in the scientific community over the last few years. Several in-depth studies of patients with HCC have established associations between particular microbiome compositions and the development of HCC. For example, genera, such as *Bacteroides*, *Phascolarctobacterium*, *Enterococcus*, *Streptococcus*, *Gemella*, *Bilophila,* are increased in patients with HCC from NAFLD and cirrhosis compared to healthy controls [43]. On the contrary, *Akkermansia*, *Bifidobacterium*, *Dialister*, and *Collinsella* were found to be downregulated in these patients [43]. Other studies pertaining to cirrhosis mediated HCC have identified genera, such as *Klebsiella*, *Haemophilus, Escherichia*-*Shigella*, *Proteus*, *Subdoligranulum*, *Prevotella 2*, *Barnesiella* to be upregulated in patients with HCC, whereas *Alistipes, Phascolarctobacterium, Rumnocccus, Buchnera*, *Megamonas*, *Lachnospira*, and *Eubacterumventriosum* are downregulated [35,44]. Additional studies have identified microbial dysregulation in the setting of HCC and hepatitis B and/or alcohol. For example, three microbes are upregulated when alcohol is present: *Enterobacter cloacae, Methylorubrum populi BJ001*, and *Rothia dentocariosa* [44]. In patients with HCC with both HBV/alcohol, *Cutibacterium acnes* is upregulated, whereas in the cohort of people with HBV and nondrinkers, *Moraxella* sp was detected [44]. In the HBV-/alcohol cohort, there is an up-regulation of *Methylorubrum populi* and a downregulation of *Acidovorax ebreus TPSY* [44]. Interestingly, there appears to be a difference in microbial profile in patients with HCC depending on the etiology of HCC. One study which analyzed patients with HBV–HCC vs. non-HBV/HCV HCC revealed that the species richness of fecal microbiota of HBV–HCC patients was much higher. Furthermore, patients with non-HBV/HCV–HCC harbored fewer potential anti-inflammatory bacteria and more pro-inflammatory bacteria [35]. Another study supported this notion of intestinal dysbiosis by demonstrating that intestinal microbial imbalance was more likely in patients with liver cirrhosis induced HCC compared to HBV/HCV–HCC [45]. Further data suggested that both heavy alcohol use and HBV may utilize the microbiome to promote the development of HCC, however, only HBV could downregulate bacteria that may promote stem cell function [45,46].

With regard to microbial impact on inflammatory and pro-carcinogenic pathways, multiple pathways have recently been identified. In patients who are HBV+, *Staphylococcus epidermidis, Acinetobacter baumanni, Methylorubrum populi*, and *Acinetobacter calcoaceticus* were strongly associated with the upregulation of ATF2, AKT, and PIGF, as well as the downregulation of P53 [44]. Furthermore, the increased activity of the WNT pathway and the PTEN/AKT pathway has been connected to the upregulation of a number of microbes in the HBV+ HCC, though most of the microbes were also related to increased activity of stem cell pathways, specifically YAP and embryonic stem cell differentiation pathways [47].

**Figure 3:**
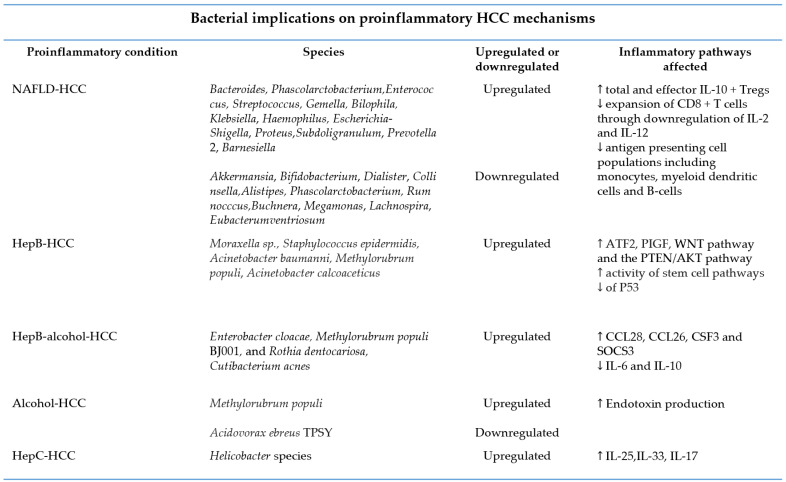
Inflammatory implications of bacteria on immune cells and metabolic pathways in conditions associated with HCC. ↑=upregulated, ↓ = downregulated.

The microbiome also has direct implications on inflammatory cytokines in the setting of HCC. Ex vivo data indicate that the gut microbiota in patients with NAFLD–HCC promotes the expansion of total and effector IL-10 + Tregs with reduced expansion of CD8 + T cells through downregulation of IL-2 and IL-12 [48]. These data suggest that the microbiota may play a direct role in immunosuppression conducive to a pro-neoplastic milieu. Moreover, bacteria from the NAFLD–HCC phenotype were shown to attenuate antigen presenting cell populations, including monocytes, myeloid dendritic cells, and B-cells compared to NAFLD-cirrhosis patients [48]. In the HBV+/alcohol cohort, cytokines, including CCL28, CCL26, CSF3, and SOCS3, were increased as a consequence of the upregulation of microbes [46]. The presence of other microbes, however, led to the suppression of other distinct cytokines, such as IL6 and IL10 [46] (Figure 3).

The microbiome also leads to hepatic inflammation via a phenomenon known as the “leaky gut”. As we have explored in our previous work regarding inflammatory mechanisms in colorectal cancer, intestinal microbiota dysbiosis leads to an array of deleterious effects on the intestinal mucosa, including intestinal barrier disruption which, in turn, leads to invasion of pathogens and harmful metabolites which are pro-inflammatory and pro-carcinogenic [49]. Given that the liver is the first organ to receive contents absorbed through the intestines via portal circulation, many of the same pathogens which lead to a pro-inflammatory milieu in the intestines also lead to inflammation in the liver. For example, hepatic exposure to gut-derived microbiota-associated molecular patterns (MAMPs), such as lipopolysaccharide (LPS), promote hepatic inflammation, fibrosis, proliferation, as well as activation of anti-apoptotic signals [50]. Specifically, these MAMPs play a significant role in the activation of host pattern recognition receptors (PRRs) in the liver, especially the Toll-like receptor family (TLRs) [51]. Various types of hepatic cells express TLR4 in particular, including Kupffer cells, hepatic stellate cells (HSCs), and endothelial cells. In experiments involving animal models, TLR4 expression on liver cells was implicated in the promotion of hepatocarcinogenesis and fibrogenesis [21]. Similar to colorectal cancer, it has been found that the TLR4-LPS pathway promotes the HCC microenvironment through activation of pro-inflammatory NF-κB, specifically through MyD88 pathway [52]. More recent data have suggested a more specific mechanism of LPS induced hepatocytic inflammation via exosomal release of HMGB1, a nuclear protein implicated in cellular death and tissue inflammation [53]. Furthermore, the activation of TLR4 by LPS also induces the epithelial–mesenchymal transition, leading to enhanced invasive capacity of HCC cells [16].

## 4. Role of Microbiome on EMT

The concept of epithelial mesenchymal transition (EMT) has proved to be important in the understanding of pathobiology of many disease processes. Although EMT is paramount in normal physiologic processes, such as wound repair and tissue remodeling, it has also been identified in more pathologic processes, such as carcinogenesis [54,55]. EMT is largely characterized by decreased expression of epithelial tight junction proteins, such as E-cadherin and ZO-1, with subsequent cytoskeletal re-arrangement, basement membrane degradation, and increased expression of mesenchymal proteins, such as vimentin, fibronectin, Snail, and Twist [56,57]. EMT has been shown to be linked to inflammatory pathways, such as IKK and MAPK [58]. In the IKK pathway, NFκB has been linked to regulation of ZEB2, a regulator of EMT whereas in the MAPK pathway, p38 MAPK has been linked to TGFβ-induced EMT [58,59]. In HCC, TGFβ enacts pro-oncogenic functions, promoting EMT and cancer dissemination [60]. With regard to the role of the microbiome on EMT, bacterial components, such as LPS and flagellin, have been linked to MAPK signaling and upregulation of NFκB [61]. It has been shown that LPS directly activates JNK/MAPK signaling in HCC cells via TLR4 to enhance their invasive ability and induce the epithelial mesenchymal transition (EMT). It is felt that through chronic microbial induced inflammation, persistent activation of these signaling cascades promotes EMT and subsequent development of fibrogenesis and carcinogenesis. For example, *Helicobacter pylori (H. pylori)* has been linked to a pro-tumorigenic environment through acceleration of liver fibrosis in the setting of CCL4 [62]. In a murine model, *H. pylori* infection has been shown to enhance the effect of CCL4 on activation of MAPK and p53 pathways, as well as enhancement of Bax and proliferating cell nuclear antigen expression [62]. Furthermore, in comparison with the CCL4-only treatment group, the number of mRNAs encoding inflammatory cytokines was highly augmented in livers of patients with *H. pylori* when treated with CCL4 [62]. Based on in vitro studies using hepatic stellate cells (HSCs), it was demonstrated that *H. pylori* increased the proliferation of HSCs, which was further enhanced by the presence of TGF-β1 [63]. The presence of TGF-β1 further induced the translocation of NF-κB into the nucleus upon treatment with *H. pylori*, leading to activation of inflammatory cascades [63]. E Coli has also been shown to promote EMT via MAPK and PIK3 pathway manipulation [64]. Activation of these pathways leads to downstream upregulation of HIF-1α protein expression and subsequent loss of both E-cadherin and cytokeratin 18 and an increase in fibronectin expression [65].

In addition to bacterial microbes, viruses have also been implicated in EMT induction. For example, one study found that when the gene of a hepatitis B secretory protein known as X protein was transfected into hepatocytes, a change from epithelial to spindle-like morphology occurred along with increased invasive abilities in the cells [66]. Hepatitis C virus (HCV) was found to impact the TGF-β/Smad axis via HCV core protein; with core protein expression directly linked to EMT in murine and human hepatocyte cell lines [67,68]. Interestingly, the EMT was reversed by addition of TGF-β inhibitor, further supporting the crucial role of this inflammatory cytokine on EMT and carcinogenesis [69].

In summary, the study of the microbial signatures involved in EMT formation not only helps to uncover discrete inflammatory pathophysiologic mechanisms of HCC progression, but also may help to provide an avenue of personalized therapies for HCC based on risk factors (i.e., patients with *H. pylori* mediated inflammation vs. HBV/HCV).

## 5. Microbial Metabolites

Over the last decade, there has been a high volume of research into the role of microbial metabolites in human health and disease. Microbial metabolites have been found to play profound roles on inflammatory pathways within the tumor micro-environment and carcinogenesis [70]. With respect to HCC formation, there are multiple metabolites with inflammatory implications, such as bile acids (BA). A high level of BAs can cause damage to hepatic DNA, which may result in carcinogenesis through the stimulation of tumor suppressor genes and oncogenes [71]. One major BA sensing receptor, FXR has been shown to be downregulated in human HCC cells [72]. In another study, mice lacking the FXR gene spontaneously developed HCC, which was associated with BA mediated inflammation, and activation of the Wnt/β-catenin signaling pathway [25,73]. Using a NASH-associated HCC model, it was demonstrated that the accumulation of secondary BAs from bacteria led to hepatic inflammation, thereby promoting mTOR signaling in hepatocytes and contributing to enhanced carcinogenesis [74]. This accumulation of bile acids was attenuated by antibiotics, suggesting role of bacteria in the conversion of primary to secondary bile acids. Interestingly, emerging evidence suggests differences between male and female sexes and bile acid profiles in HCC. In a murine NASH–HCC model, it was found that male mice had increased hepatic retention of BAs and decreased expression of tumor suppressive miRNAs when compared to female mice [75]. Furthermore, metagenomic analysis showed differences in gut microbiota involved in BA metabolism between normal male and female mice. There were also observed differences in microbial profiles [75]. *Firmicutes* were decreased significantly in female mice, in contrast to a significantly increased Firmicutes population in male mice. The Firmicutes to Bacteroidetes ratio was markedly increased in male mice and decreased significantly in female mice [75].

Although bile acids have a well-established pro-inflammatory role in the liver, some evidence suggests that they possess antitumor properties. In an animal model, it was found that primary bile acids increased expression of CXCL16, a ligand which positively regulates natural killer T cells [76]. Furthermore, it was found that antibiotic treatment led to an increase in NKT cells and a decrease in tumor growth [76]. Moreover, the administration of secondary bile acids was correlated with a reversal of NKT accumulation and a corresponding increase in tumor size. In another experiment involving humans, researchers found a correlation between primary bile acid cheno-deoxycholic acid (CDCA) levels and CXCL16 expression in non-tumor liver tissue from patients with primary liver cancer, but a negative correlation with secondary bile acid glycolithocholate (GLCA), suggesting that CDCA had a direct role in preventing tumor expansion [76]. In addition, conjugated secondary 12α-OH BAs have been found to significantly increase the liver fibrosis markers α-SMA, TGF-ß, COL I, and PDGF via TGR5 mediated p38MAPK and ERK1/2 signaling [77]. Although these studies suggest that secondary bile acids have the potential to be proinflammatory and protumorigenic, more studies are necessary to elucidate this idea. For example, the secondary bile acid ursodeoxycholic acid is well known for its anti-inflammatory properties in primary biliary cholangitis. Some studies have even suggested ursodeoxycholic acids anti-inflammatory implications in colitis [78]. Given this conflicting effect of secondary bile acids, more metabolomic studies which are able to identify and stratify subtypes of secondary BAs based on pro- or anti-inflammatory potential are necessary.

Short chain fatty acids (SCFAs) have also been implicated in microbiota mediated inflammation in hepatocytes. The fermentable fiber inulin induces HCC in mice in a microbiota-dependent manner, but not in germ-free or antibiotic-treated mice [79]. Furthermore, inulin was shown to lead to neutrophil infiltration and hepatic inflammation. Interestingly, these effects were reversed by decreasing SCFA producing bacteria or by eliminating fermentable fibers [79]. Another study in NAFLD patients revealed increased levels of SCFAs in the fecal microbiota, further supporting the notion of an inflammatory role given that NAFLD patients are known to have lobular inflammation [80]. Furthermore, fermentable fiber has been linked to altered gut microbiota and elevation of hepatic bile acids concentrations; which are associated with hepatic inflammation [81].

**Figure 4:**
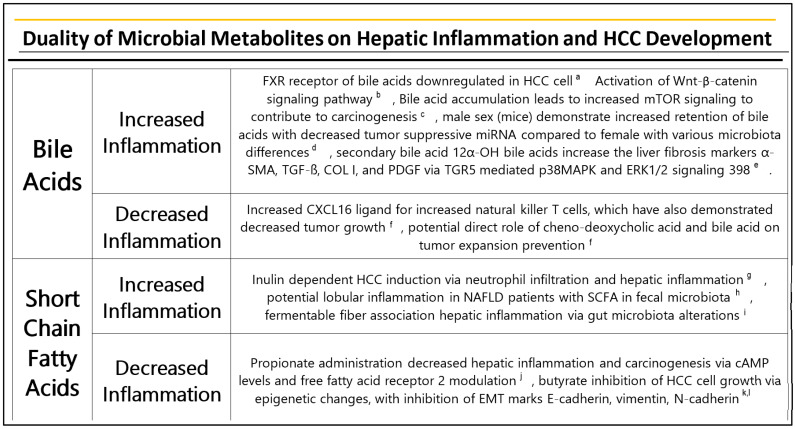
Effect of bile acids and short chain fatty acids on upregulation and downregulation of inflammatory processes which lead to HCC. ^a^ Su et al., 2012 [74], ^b^ Zhang et al., 2012 [75], ^c^ Yamada et al., 2018 [76], ^d^ Xie et al., 2017 [77], ^e^ Xie et al., 2021 [78], ^f^ Ma et al., 2018 [79], ^g^ Singh et al., 2018 [80], ^h^ Lee et al., 2020 [82], ^i^ Janssen et al., 2017 [83], ^j^ Gupta et al., 2019 [84], ^k^ Wang et al., 2013 [85], ^l^ Matsui-Yuasa et al., 2016 [86].

SCFAs have also been shown to have protective effects against hepatocarcinogenesis. For example, one study showed evidence of decreased hepatic inflammation and carcinogenesis from administration of propionate [82]. Specifically, the propionate levels were dependent on cyclic adenosine monophosphate (cAMP) level and free fatty acid receptor 2 modulation [82]. Another SCFA, butyrate was found to have inhibitory effects on the growth of HCC cells via epigenetic changes. Furthermore, many EMT markers such as E-cadherin, vimentin, and N-cadherin were also inhibited by butyrate, suggesting a multimodal anticarcinogenic effect of this compound [83,84] (Figure 4).

## 6. Role of the Microbiome on the Immune System and Immunotherapy

**Figure 5:**
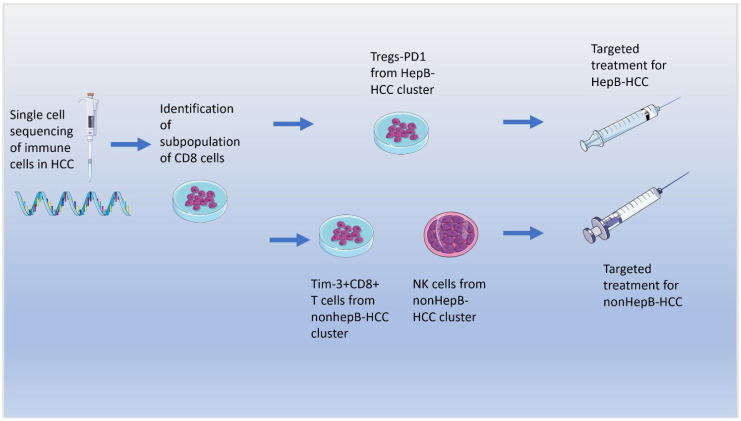
Schematic depiction of single cell technology and how it can potentially be used for targeted, individualized treatment of HCC based on immune cell, and HCC risk factor phenotype.

Recent evidence has suggested the microbiome plays a crucial role on immune system modulation for multiple disease processes. For example, the FMT from mice with colorectal cancer to healthy mice was found to increase the expression of an array of pro-tumorigenic and pro-inflammatory cytokines, as well as polarization of tumor-promoting macrophages [87]. With respect to HCC, there have been great advances in understanding of immune mediators and their role in HCC formation through recent technological breakthroughs, including single cell, as well as RNA-seq analyses. In fact, using these technologies, HCC was found to be the most heterogenous cancer of all solid tumor cancers with differences on intertumoral and intratumoral levels [88,89]. This heterogeneity has led to improved subclassification of patients based on molecular analysis [90]. Single-cell RNA sequencing (scRNA-seq) studies of immune cells in HCC tumors have recently revealed a subpopulation of CD8^+^ T cells which expressed high levels of markers, such as cytotoxic T lymphocyte-associated protein 4 (*CTLA4*), *PDCD1* (also called PD1), and *HAVCR2* (also called TIM3) [91]. Another study which focused on the immunological profile between patients with hep-B HCC vs. non-hep-B HCC revealed that tumor infiltrating lymphocytes of hep-B HCC clusters had a high abundance of T regs and elevated expression of PD-1 expression, whereas non-hep-B HCC clusters contained Tim-3^+^CD8^+^ T cells and NK cells [92]. This study suggests a potential role of personalized immunotherapy through the targeting of different therapeutic biomarkers based on the subtype of hepatocellular carcinoma. With respect to the microbial implications on immune system modularity, a recent study which examined patients who were receiving anti PD-1 therapy for unresectable HCC revealed that a higher abundance of the taxa *Lachnospiraceae* bacterium-GAM79 and *Alistipes sp Marseille-P5997* was associated with improved clinical response, as well as overall survival compared to patients with lower abundance of these taxa [93] (Figure 5).

Other studies have identified *Bifidobacterium* and *Bacteroides* in mouse gut microbiota to be correlated with tumor suppression by inhibition of PD-1 and CTLA-4 blockade, respectively [94,95]. Despite this promising evidence, the number of studies examining microbial implications on immune system modulation in HCC is still limited. Much of the evidence is focused on non-HCC malignancies. For example, one RNA-seq study identified a prominent co-inhibitory signal in T-cells and antigen presenting cells via the *TIGIT*–*NECTIN2* (Nectin Cell Adhesion Molecule 2) [96]. Furthermore, there was a statistically significant difference identified between TIGIT and NECTIN2 expression in Hep-B-HCC, but not in non-HCC-Hep-B cirrhotic livers [96]. *Fusobacterium nucleatum* was found to directly interact with TIGIT through the FAP2 protein, with subsequent inhibition of NK cells which have significant anti-tumor properties [97]. However, this study was limited to colorectal, thymoma and leukemic cells, necessitating further research into the potential role in HCC.

In conclusion, the microbiome plays a tremendous role in the inflammatory pathogenesis of HCC. With a deeper understanding of these implicated mechanisms, not only do we have a better understanding of the pathogenesis of HCC, but we also create a foundational knowledgebase from which we can derive potential biomarkers and therapeutic targets.

## Data Availability

Not applicable.
